# The relationship between tobacco and breast cancer incidence: A systematic review and meta-analysis of observational studies

**DOI:** 10.3389/fonc.2022.961970

**Published:** 2022-09-15

**Authors:** Yujing He, Yuexiu Si, Xiangyuan Li, Jiaze Hong, Chiyuan Yu, Ning He

**Affiliations:** ^1^ The Second Clinical Medical College, Zhejiang Chinese Medical University, Hangzhou, China; ^2^ School of Basic Medical Sciences, Zhejiang Chinese Medical University, Hangzhou, China; ^3^ Department of Tumor High-intensity focused ultrasound (HIFU) Therapy, HwaMei Hospital, University of Chinese Academy of Sciences, Ningbo, China

**Keywords:** breast cancer, active smoking, passive smoking, incidence, meta-analysis selection criteria

## Abstract

**Background:**

The effect of tobacco on breast cancer (BC) is controversial. The purpose of this study was to investigate the relationship between tobacco and BC.

**Methods:**

A search was conducted in PubMed, EBSCO, Web of Science and Cochrane Library databases before February 2022. The adjusted odd ratio (OR) and corresponding 95% confidence interval (CI) were used to examine the relationship between active or passive smoking and BC risk.

**Results:**

A total of 77 articles composed of 2,326,987 participants were included for this meta-analysis. Active (OR=1.15, 95% CI=1.11-1.20, p<0.001) and passive (OR=1.17, 95% CI=1.09-1.24, p<0.001) smoking increased the risk of BC in the female population, especially premenopausal BC (active smoking: OR=1.24, p<0.001; passive smoking: OR=1.29, p<0.001), but had no effect on postmenopausal BC (active smoking: OR=1.03, p=0.314; passive smoking: OR=1.13, p=0.218). Active smoking increased the risk of estrogen receptor-positive (ER+) BC risk (OR=1.13, p<0.001), but had no effect on estrogen receptor-negative (ER-) BC (OR=1.08, p=0.155). The risk of BC was positively associated with the duration and intensity of smoking, negatively associated with the duration of smoking cessation. Active smoking increased the risk of BC in the multiparous population (OR=1.13, p<0.001), but had no effect on the nulliparous population (OR=1.05, p=0.432), and smoking before the first birth (OR=1.22, 95% CI=1.17-1.27) had a greater impact on the risk of BC than smoking after the first birth (OR=1.08, 95% CI=1.04-1.12).

**Conclusion:**

Smoking (active and passive) increased the risk of BC in women. The effect of smoking on BC was influenced by smoking-related factors (duration, intensity, years of quitting), population-related factors (fertility status), and BC subtypes.

**Systematic Review Registration:**

identifier CRD42022322699.

## Introduction

Breast cancer (BC) is the most common cancer in women worldwide (1). As a heterogeneous disease, its occurrence is influenced by both endogenous factors (such as heredity (2, 3), gene mutation (4, 5)) and exogenous factors (such as reproduction (6, 7), environment (8)). It is estimated that only 5-10% of BC cases are induced by genetic factors, while the remaining 90-95% are highly related to environmental factors or specific lifestyle (9, 10). Therefore, researchers are trying to provide better preventional strategies by adjusting exposure to BC protective or risky factors (1, 11). Evidence has shown that unhealthy lifestyle and some environmental factors are harmful to women (12–14), and eliminating these factors may help reduce the morbidity and mortality rate (15, 16).

The potential role of smoking in BC risk has been under intense discussion (17, 18). Although BC is not initially thought to be a tobacco-related cancer, over the past few decades, many chemicals contained in tobacco have been investigated to be a trigger of BC, such as 4-aminobiphenyl (19, 20) and benzopyrene (21, 22). In addition, evidence of the role of active smoking (23, 24) and secondhand smoke (25, 26) in the etiology of BC is accumulating, based on adequate animal trials (27, 28) and relevant epidemiological evidence (29). Recent trends have discovered smoking as one of the potential risk factors for BC (30).

Although many studies have shown that smoking may increase the risk of BC, a review of studies over the past 30 years has found that opinions among clinical researchers are still widely divided (17, 31). Firstly, some studies [e.g. Yingsong Lin et al. (32) and Chelsea Catsburg et al. (23)] failed to observe any association between smoking and BC incidence. Secondly, the results of subgroup analyses among different studies were high inconsistent (33, 34), or even reversed, such as subgroup analyses on menstrual status and BC subtypes. Third, published meta-analyses on the topic have also not reached consistent conclusions. Although most meta-analyses on active smoking suggest that smoking increases the incidence of BC, the conclusions of subgroup analyses are inconsistent (35, 36), and the meta-analyses on passive smoking are more inconsistent (37, 38). The last relevant meta-analysis was conducted and published in 2018. As of 2021, there are 153 million adult female smokers (including smoking, secondhand, and chewing) worldwide, accounting for 12% (39) of global smokers. Therefore, based on the inconsistency of previous studies, the large smoking population and the significant disease burden caused by tobacco (40), this study aimed to investigate the relationship between smoking and BC by conducting a systematic review and meta-analysis by searching for relevant observational studies. Therefore, it can provide a preventive reference for the female group and create greater value for the society.

## Materials and methods

### Search strategy

A comprehensive search of studies investigating the association between smoking and BC was carried out before February 2022 in electronic databases of PubMed, Web of science, EBSCO, and the Cochrane Library. The complete retrieval formula that was used to identify the related studies includes: (“breast cancer” OR “breast neoplasms” OR “BC”) AND (“smoking” OR “tobacco smoke pollution” OR “tobacco use” OR “tobacco products” OR “active smoking” OR “passive smoking” OR “secondhand smoking” OR “tobacco”). The reference lists of retrieved studies and conference records were also reviewed for potentially inclusive studies. When referring to duplicate literature, the original article was included if the study was published as an abstract or an original article. Also, if a study was continuously updated and reported, only the most recent or comprehensive articles were included. This meta-analysis was conducted according to the Meta-Analysis of Observational Studies in Epidemiology (MOOSE) guidelines (41). The population, intervention, comparison, outcome, and setting (PICOS) criteria were used to describe the research question. Participants in this study were people who had not previously been diagnosed with BC, the intervention was exposure to tobacco environments, including active and passive smoking, the comparison was a non-smoker, the outcome was the incidence of BC, and the setting was observational research. This meta-analysis’s prospero registration number was CRD42022322699.

### Selection criteria

An eligible criterion was formulated. The specific criteria were as follows. Inclusion criteria: (1) all included studies are observational studies. (2) The main exposure of study was smoking including active and passive smoking, and the outcome was BC risk. (3) All studies included available data which reported the relationship between smoking and BC. Exclusion criteria: (1) the study was conducted on BC population and used mortality or recovery rate as the outcome. (2) The study was published in duplicate. (3) The study was not published in English.

### Data collection and quality assessment

A jointly agreed data collection form was used to extract all data. Information was extracted as follows: the author’s name, year of publication, study type, age, exposure assessment, number of participants, number of BC cases, number of smokers, number of non-smokers, variables adjusted in the statistical analyses, and outcomes. To ensure the objectivity and accuracy of the data, two researchers independently extracted data from each study. Disagreements were resolved by consensus or consultation with a third researcher.

The quality of each included study was evaluated by the Newcastle-Ottawa Quality Assessment Scale (NOS) checklist, a tool used for quality assessment of non-randomized studies. NOS checklist is composed of eight items classified into three aspects, including selection, comparability, and outcome. The maximum scores of this checklist were nine, and scores between seven and nine were identified to be of higher study quality.

### Objectives and endpoints

The primary objective was to explore the relationship between smoking and the incidence of BC. Secondary objectives were to explore the relationship between the incidence of BC and smoking subgroups (e.g. smoking pattern, smoking time, smoking frequency, smoking place, smoking cessation time, age of starting smoking), the relationship between smoking and BC in different populations (e.g. fertility status, menopausal status, race), and the association between smoking and different BC subtypes (e.g. estrogen receptor-positive (ER+) BC, estrogen receptor-negative (ER-) BC). The results after adjusting for relevant confounding factors were used consistently for the processing of relevant data from the included articles.

### Statistical analysis

The Stata software version 12 (StataCorp, College Station, Texas, USA) was used to analyze the data. The confidence interval (CI) of odd ratio (OR) was set at 95% to examine the relationship between smoking and BC risk. Heterogeneity of included studies was tested by Q statistic and I^2^ statistic to quantitatively assess inconsistency. For statistical results, values of p<0.10 and I^2^>50% were considered to be representative of having statistically significant heterogeneity. Based on the heterogeneity of smoking intensity, smoking duration, race, BC subtype, etc. in different studies, in order to improve the reliability of the results, the random effects model was uniformly used in this study. When more than ten studies were included, sensitivity analysis and publication bias test were performed to evaluate the stability and reliability of their results. Publication bias was evaluated by the Begg’s test. Results with P-values less than 0.05 were considered to be statistically significant.

## Results

### Literature search

**Figure 1:**
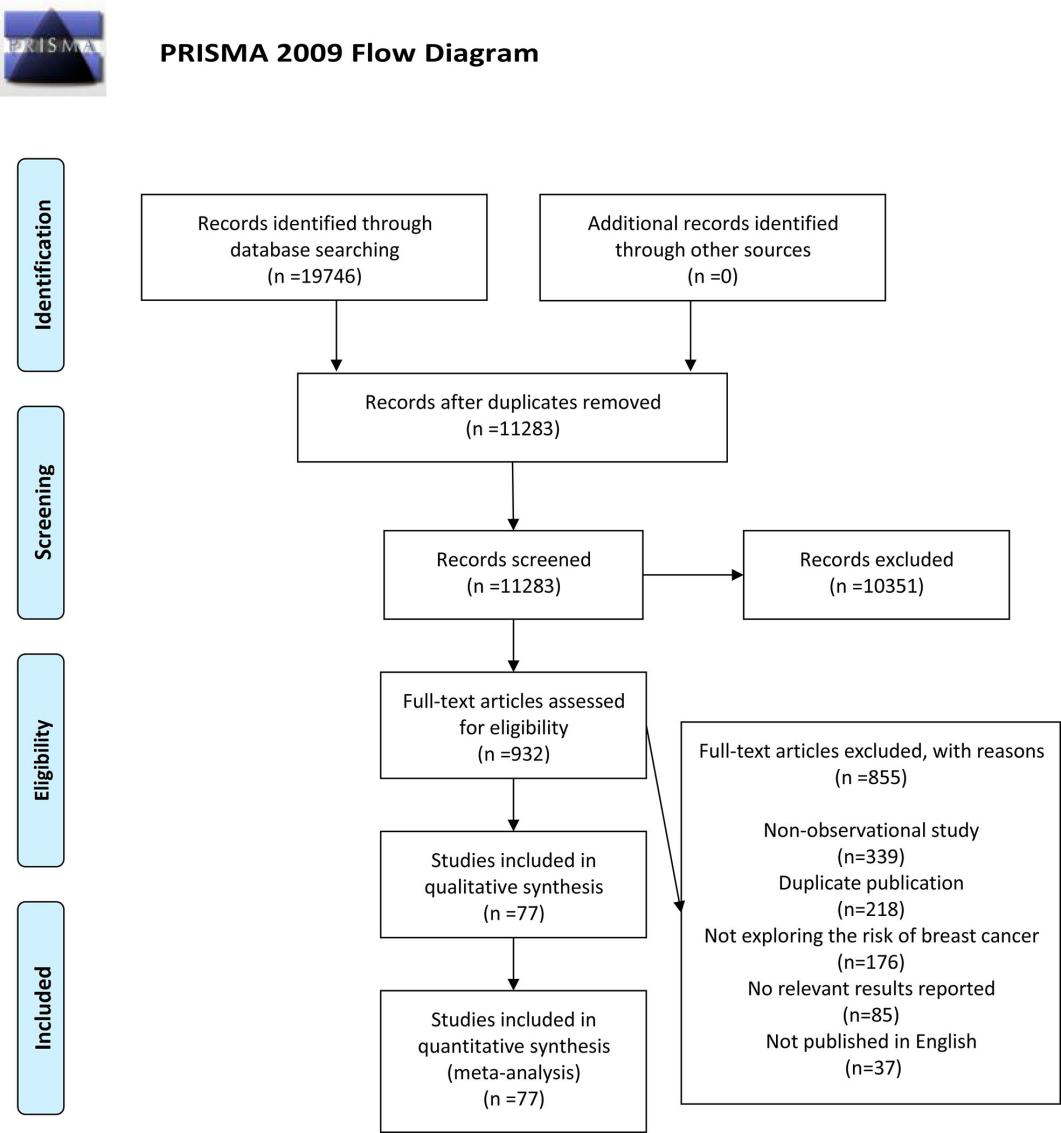
A schematic flow for the selection of articles included in this meta-analysis.

A total of 19,746 relevant articles were identified based on retrieval formula described in the methods section by initial search in PubMed, EBSCO, Web of Science, and Cochrane Library database. No additional records were identified through other sources. A total of 8,463 duplicate articles were deleted, and 11,283 articles were excluded due to the title or abstract. The remaining 932 articles were reviewed through full-text. Among them, 855 articles were eliminated because of being non-observational study (n=339), duplicate publication (n=218), not exploring the risk of BC (n=176), no relevant results reported (n=85), and not published in English (n=37). Eventually, 77 articles (13, 32–34, 42–115) composed of 2,326,987 participants were selected for this meta-analysis. The detailed search and study selection process was shown in **Figure 1**.

### Characteristic of studies

Of the 77 included studies, 24 were cohort studies (2,138,338 participants and 55,703 BC cases), 53 were case-control studies (188,649 controls and 58,859 BC cases). The participants in the two studies included men and women, and the rest were women. All studies were published between 1988 and 2022, with follow-up periods ranging from 6 to 24.6 years. Regarding age at recruitment, eight studies did not set the upper age limit, four studies did not set a lower age limit and four studies did not report the requirement for age. Among them, 30 studies were conducted in America, 24 were in Asia, 22 were in Europe, and 1 was in Oceania. Fifty-six studies investigated the association between active smoking and BC risk, 39 investigated the association between passive smoking and BC risk. The number of smokers (included active and passive smokers) was 1,326,603 in cohort studies and 108,175 in case-control studies. In order to collect data and evaluate relevant exposure factors, 59 studies chose questionnaire, 9 studies chose interview, and 9 studies chose questionnaire combined with interviews. In addition, the adjustment of potential confounding factors varied in different studies. Most of the adjustment parameters were age, body mass index (BMI), family history of BC, total energy intake, alcohol consumption, number of births, and physical activity. The characteristics of the included studies were shown in **Table 1** and **Supplementary Table 1**.

**Table 1:** Characteristics of included observational studies in the meta-analysis.

Author, year	Country	Median follow-up time (years)	Age at recruitment (year)	Median age at time of analysis (years)	No. of BC cases	No. of participants	Study Type
Vatten LJ, 1990	Norway	12	35-51	NA	242	24,617	Cohort study
Bennicke K, 1995	Denmark	NA	15-92	45.0	230	3,240	Cohort study
Calle EE, 1994	America	6	30-75	56.0	880	604,412	Cohort study
Goodman MT, 1997	Japan	8.31	30-85	64.5	161	22,200	Cohort study
Nishino Y, 2001	Japan	9	>40	56.6	67	9,675	Cohort study
Hanaoka T, 2005	Japan	9	40-59	49.0	180	21,805	Cohort study
Olson JE, 2005	America	14	55-69	62.0	2,017	37,105	Cohort study
Lin Y, 2005	Japan	7.8	40-79	57.0	208	34,410	Cohort study
Pirie K, 2008	United Kingdom	6.3	50-64	57.0	2,518	210,647	Cohort study
Reynolds P, 2009	America	8	>35	53.0	1,754	57,523	Cohort study
Xue F, 2010	America	24.6	30-55	58.0	8,772	121,700	Cohort study
Luo J, 2011	America	10.3	50-79	62.0	3,520	79,900	Cohort study
Rosenberg L, 2013	America	14	21-69	37.0	1,377	59,000	Cohort study
Dossus L, 2014	France	11	35-65	58.0	9,822	322,988	Cohort study
Catsburg C, 2015	Canada	22.1	40-59	52.0	6,549	89,835	Cohort study
Wada K, 2015	Japan	10	>35	53.0	543	15,719	Cohort study
White AJ, 2017	America	6.4	35-74	54.9	1,843	50,884	Cohort study
van den Brandt PA, 2017	Netherlands	NA	55-69	59.0	2,526	62,573	Cohort study
Jones ME, 2017	United Kingdom	7.7	>16	47.0	1,815	102,927	Cohort study
Gram IT, 2019	America	16.7	45-75	62.0	4,230	67,313	Cohort study
Heberg J, 2019	Denmark	18.8	>44	56.0	1,407	16,106	Cohort study
Zeinomar N, 2019	America	10.4	18-79	46.7	1,009	17,435	Cohort study
Botteri E, 2021	Sweden	9.5	30-49	40.0	1,848	29,930	Cohort study
Gram IT, 2022	Norway	19.8	34-70	49.8	2,185	76,394	Cohort study
Kato I, 1992	Japan	NA	20-75	48.0	908	1,816	Case-control study
Field NA, 1992	America	NA	20-79	NA	1,617	3,234	Case-control study
Pawlega J, 1992	Poland	NA	35-75	52.0	127	377	Case-control study
Chu SY, 1990	America	NA	20-54	45.0	4,134	8,351	Case-control study
Schechter MT, 1989	Canada	NA	40-59	NA	254	1,061	Case-control study
Adami HO, 1988	Sweden, Norway	NA	<45	37.0	422	949	Case-control study
Hirose K, 1995	Japan	NA	20-80	49.0	1,186	24,349	Case-control study
Smith SJ, 1994	United Kingdom	NA	<36	NA	755	1,502	Case-control study
Braga C, 1996	Italy	NA	20-74	56.0	2,569	5,157	Case-control study
Ranstam J, 1955	United Kingdom	NA	25-59	NA	998	1,996	Case-control study
Morabia A, 1998	Switzerland	NA	30-74	53.0	242	1,301	Case-control study
Tung HT, 1999	Japan	NA	29-85	51.6	376	806	Case-control study
Johnson KC, 2000	Canada	NA	25-74	43.0	2,317	4,755	Case-control study
Marcus PM, 2000	America	NA	20-74	NA	864	1,654	Case-control study
Ueji M, 1998	Japan	NA	26-69	48.0	145	385	Case-control study
Lash TL, 2002	America	NA	40-85	65.0	615	1,281	Case-control study
Kropp S, 2002	Germany	NA	<50	43.0	468	1,561	Case-control study
Liu L, 2000	China	NA	24-55	41.0	186	372	Case-control study
Shrubsole MJ, 2004	China	NA	25-64	47.0	1,013	2,130	Case-control study
Alberg AJ, 2004	America	NA	NA	NA	110	223	Case-control study
Gammon MD, 2004	America	NA	24-98	56.0	1,356	2,739	Case-control study
Manjer J, 2004	Sweden	NA	NA	59.0	260	801	Case-control study
Bonner MR, 2005	America	NA	35-79	51.0	1,166	3,271	Case-control study
Metsola K, 2005	Finland	NA	44-77	55.0	483	965	Case-control study
Mechanic LE, 2006	America	NA	NA	NA	2,311	4,333	Case-control study
Ha M,2007	America	NA	22-92	37.5	906	12,372	Case-control study
Roddam AW, 2007	United Kingdom	NA	36-45	41.0	639	1,279	Case-control study
Slattery ML,2008	America	NA	>50	NA	1,183	2,266	Case-control study
Rollison DE, 2008	America	NA	40-79	63.0	287	598	Case-control study
Young E, 2009	America, Canada	NA	25-75	55.0	6,235	12,768	Case-control study
Ahern TP, 2009	America	NA	<75	59.0	557	989	Case-control study
Conlon MS, 2010	Canada	NA	25-75	55.9	347	1,122	Case-control study
De Silva M,2010	Sri Lanka	NA	30-64	48.0	100	303	Case-control study
Sezer H, 2011	Turkey	NA	35-60	54.0	172	555	Case-control study
Hu M, 2013	China	NA	25-75	46.7	196	407	Case-control study
Gao CM, 2013	China	NA	30-65	50.0	669	1,351	Case-control study
McKenzie F, 2013	New Zealand	NA	NA	NA	1,799	4,339	Case-control study
Ilic M, 2013	Serbia	NA	30-75	60.0	191	382	Case-control study
Kawai M, 2014	America	NA	20-44	35.0	1,920	2,858	Case-control study
Tong JH, 2014	China	NA	>18	49.0	312	624	Case-control study
Pimhanam C, 2014	Thailand	NA	17-76	45.0	444	888	Case-control study
Li B, 2015	China	NA	25-70	46.0	877	1,767	Case-control study
Connor AE, 2015	Spain	NA	25-70	7026.0	2,889	7,917	Case-control study
Hara A, 2017	Japan	NA	35-85	55.0	511	1,038	Case-control study
Butler EN, 2016	America	NA	20-64	51.0	1,808	3,372	Case-control study
Park SY, 2016	America	NA	20-75	43.0	5,791	23,167	Case-control study
Strumylaite L, 2017	Lithuania	NA	28-90	60.0	449	1,379	Case-control study
Dianatinasab M, 2017	Iran	NA	35-65	49.0	526	1,052	Case-control study
Ellingjord-Dale M, 2017	Norway	NA	50-69	58.0	4,420	28,700	Case-control study
Regev-Avraham Z, 2018	Israel	NA	30-70	52.8	137	411	Case-control study
Godinho-Mota JCM, 2019	Brazil	NA	30-80	41.0	197	542	Case-control study
Alsolami FJ, 2019	Saudi Arabia	NA	45-75	57.0	214	432	Case-control study
Baset Z, 2021	Afghanistan	NA	>30	45.8	201	402	Case-control study

NA, not available; BC, breast cancer.

### Overall effect of active smoking

Fifty-six studies recorded data about active smoking in female population that was inducing BC. Studies had shown that women who actively smoked had a significantly higher incidence of BC than those who had never actively smoked (OR=1.15, 95% CI=1.11-1.20, p<0.001, I^2 =^ 54.9%). Among them, current active smoking (OR=1.12, 95% CI=1.08-1.16, p=0.007, I^2 =^ 40.1%) and former active smoking (OR=1.09, 95% CI=1.06-1.12, p<0.001, I^2 =^ 33.3%) had a significantly increase on the incidence of BC, but current active smoking increased the incidence of BC more than former active smoking. In other words, active smoking is a risk factor for women, and the population who is still active smoking is under more risk than the population who quit smoking after active smoking. In addition, cohort studies (OR=1.13, p<0.001) and case-control studies (OR=1.19, p<0.001) had consistently concluded that active smoking increases the risk of BC in women. The detailed data was contained in **Table 2**.

**Table 2:** Effects of active smoking on breast cancer incidence.

Subgroup analysis	No. ofstudies	OR	95%CI	*p*	Heterogeneity (I^2^) (%)
Ever active smoking	56	1.15	1.11-1.20	*<0.001*	54.9
Current	39	1.12	1.08-1.16	*0.007*	40.1
Former	42	1.09	1.06-1.12	*<0.001*	33.3
Cohort study	17	1.13	1.07-1.18	*<0.001*	72.6
Case-control study	39	1.19	1.12-1.26	*<0.001*	31.9
Premenopausal BC	23	1.24	1.17-1.32	*<0.001*	6.2
Postmenopausal BC	25	1.03	0.97-1.10	0.314	30.8
Smoking duration					
<20 years	38	1.06	1.03-1.09	*<0.001*	0
20-30 years	36	1.15	1.10-1.19	*<0.001*	27.8
30-40 years	20	1.15	1.10-1.20	*<0.001*	5.7
>40 years	13	1.22	1.13-1.31	*<0.001*	40.8
Smoking intensity					
<10 cigarettes per day	35	1.06	1.03-1.10	*0.001*	13.3
10-20 cigarettes per day	38	1.19	1.14-1.25	*<0.001*	30.4
20-30 cigarettes per day	29	1.16	1.11-1.22	*<0.001*	30.2
>30 cigarettes per day	4	1.18	1.07-1.31	*0.001*	9.4
Pack-years smoked					
<10 years	31	1.05	1.01-1.08	*0.005*	5.5
10-20 yeasr	36	1.11	1.08-1.15	*<0.001*	0.9
20-40 yeasr	29	1.21	1.17-1.27	*<0.001*	17.8
>40 yeasr	12	1.17	1.11-1.23	*<0.001*	0
Age started smoking					
< 16 years	25	1.11	1.07-1.15	*<0.001*	0
17-19 years	34	1.16	1.12-1.20	*<0.001*	9.2
>20 years	33	1.08	1.04-1.11	*<0.001*	16.5
Years since quitting					
<10 years	18	1.27	1.15-1.41	*<0.001*	74.2
10-20 yeasr	18	1.05	1.00-1.09	*0.046*	5.0
>20 yeasr	11	1.01	0.97-1.06	0.552	0
Fertility status					
Multiparous population	6	1.13	1.07-1.20	*<0.001*	0
Nulliparous population	6	1.05	0.92-1.20	0.432	0
Active smoking before first birth	24	1.22	1.17-1.27	*<0.001*	9.4
<5 years before first birth	13	1.06	1.01-1.11	*0.023*	0
>5 years before first birth	21	1.24	1.14-1.35	*<0.001*	49.9
Active smoking after first birth	22	1.08	1.04-1.12	*<0.001*	0
<10 years after first birth	7	1.00	0.93-1.09	0.922	19.1
>10 years after first birth	10	1.06	0.99-1.14	0.077	48.8
BC subtypes					
ER+ BC	6	1.13	1.08-1.18	*<0.001*	0
<10 years smoking	5	0.99	0.90-1.09	0.870	30.0
>10 years smoking	13	1.14	1.04-1.25	*0.007*	49.6
<10 cigarettes per day	7	1.08	1.00-1.17	*0.041*	25.9
>10 cigarettes per day	7	1.18	1.06-1.32	*0.002*	62.7
ER- BC	6	1.08	0.97-1.19	0.155	0
<10 years smoking	5	1.02	0.91-1.16	0.699	0
>10 years smoking	13	1.08	0.98-1.18	0.105	0
<10 cigarettes per day	13	0.97	0.87-1.08	0.603	0
>10 cigarettes per day	13	1.18	1.00-1.39	*0.049*	53.5

OR, odd ratio; CI, confidence interval; ER, estrogen receptor; PR, progesterone receptor; BC, breast cancer.

### Menopausal status

The correlation between smoking and BC is affected by menopausal status. Related data were available in 23 studies with premenopausal BC and 25 with postmenopausal BC. The analysis showed that active smoking increases the incidence of premenopausal BC (OR=1.24, 95% CI=1.17-1.32, p<0.001, I^2 =^ 6.2%), but had no effect on postmenopausal BC (OR=1.03, 95% CI=0.97-1.10, p=0.314, I^2 =^ 30.8%) with slight heterogeneity. The detailed data was contained in **Table 2**.

### Smoking duration

Years were used to measure smoking duration in this study. The related data were divided into ‘<20 years group’, ‘20-30 years group’, ‘30-40 years group’, and ‘>40 years group’ according to the most studies. The results showed that women who smoked for less than 20 years (OR=1.06, p<0.001), 20-30 years (OR=1.15, p<0.001), 30-40 years (OR=1.15, p<0.001), and more than 40 years (OR=1.22, p<0.001) had a higher incidence of BC than those without smoking history. The incidence of BC was positively correlated with smoking duration. The detailed data was contained in **Table 2**.

### Smoking intensity

Cigarettes per day were used to measure smoking intensity in this study. The data is grouped by 10 cigarettes per day, 20 cigarettes per day, and 30 cigarettes per day. Subgroup analysis showed smoking which less than 10 cigarettes per day (OR=1.06, p=0.001), between 10-20 cigarettes per day (OR=1.19, p<0.001), between 20-30 cigarettes per day (OR=1.16, p<0.001), and more than 30 cigarettes per day (OR=1.18, p=0.001) increased the incidence of BC with statistical significance. The incidence of BC increased with the increase of smoking intensity. The detailed data was contained in **Table 2**.

### Pack-years smoked

Pack-years were used to simultaneously assess smoking duration and smoking intensity. Pack-years were defined as the product of the number of cigarettes smoked per day and the number of years of smoking. According to the grouping criteria of the included studies, this study divided the relevant data into ‘<10 pack-years group’, ‘10-20 pack-years group’, ‘20-40 pack-years group’, and ‘>40 pack-years group’. The analysis showed that women who smoke with less than 10 pack-years (OR=1.05, p=0.005), 10-20 pack-years (OR=1.11, p<0.001), 20-40 pack-years (OR=1.21, p<0.001), and >40 pack-years (OR=1.17, p<0.001) had a higher incidence of BC than those who had never smoked. The detailed data was contained in **Table 2**.

### Age started smoking

In this study, smoking initiation age was divided into ‘<16 years group’, ‘17-19 years group’, and ‘>20 years group’. The results suggested that active smoking, regardless of the age at which smoking started is younger than 16 years old (OR=1.11, 95% CI=1.07-1.15), between 17-19 years old (OR=1.06, 95% CI=1.12-1.20), or older than 20 years old (OR=1.08, 95% CI=1.04-1.11), would significantly increase the incidence of BC in women with slight heterogeneity. The detailed data was contained in **Table 2**.

### Years since quitting

Years of quitting smoking were used to measure the effect of smoking cessation in the participants. Data were grouped by 10- and 20-year cessation years. Subgroup analysis showed that previous smoking history remained a risk factor for BC among women who had quit smoking for less than 20 years. Among them, the harm of previous smoking history to women who quit smoking for less than 10 years (OR=1.27, 95% CI=1.15-41, p<0.001) is significantly greater than that to those who quit smoking for 10-20 years (OR=1.05, 95% CI=1.00-1.09, p=0.046). With increased time to quit smoking comes a reduction in the harm caused by previous smoking history. Previous smoking history was no longer an observable risk factor for BC in women who had quit smoking for more than 20 years (OR=1.01, 95% CI=0.97-1.06, p=0.552). The detailed data was contained in **Table 2**.

### Fertility status

Six studies explored the association between active smoking and BC in different fertility statuses. The analysis showed that active smoking can increase the risk of BC in the multiparous population (OR=1.13, 95% CI=1.07-1.20, p<0.001), but had no effect on BC in the nulliparous population (OR=1.05, 95% CI=0.92-1.20, p=0.432) without heterogeneity. The detailed data was contained in **Table 2**.

### Active smoking before/after the first birth

Regarding the relationship between active smoking and BC risk before/after the first birth, 24 studies contained data before the first birth and 22 studies contained data after the first birth. The results of the analysis showed that active smoking significantly increased the incidence of BC, regardless of whether the mother was smoking before the first birth (OR=1.22, 95% CI=1.17-1.27, p<0.001) or smoking after the first birth (OR=1.08, 95% CI=1.04-1.12, p<0.001), with slight heterogeneity. Furthermore, active smoking before the first birth had a greater impact on inducing BC than active smoking after the first birth. The detailed data was contained in **Table 2**.

Among those who actively smoked before the first birth, data were grouped by 5 years of smoking. Subgroup analysis showed that active smoking before the first birth increased the risk of BC whether the duration of smoking less than 5 years (OR=1.06, p=0.023) or more than 5 years (OR=1.24, p<0.001). There was a positive correlation between the smoking duration before the first birth and the risk of BC. Among those who have actively smoked after the first born, data were grouped by 10 years of smoking. Subgroup analysis showed that active smoking after the first birth had no effect on BC whether the duration of smoking less than 10 years (OR=1.00, p=0.922) or more than 10 years (OR=1.06, p=0.077). However, with the increase of smoking duration, active smoking had a tendency to harm the female population after the first birth by inducing BC. The detailed data was contained in **Table 2**.

### BC subtypes

Six studies examined the association between active smoking and BC subtypes. The results showed that active smoking increased the incidence of ER+ BC (OR=1.13, 95% CI=1.08-1.18, p<0.001), but had no effect on ER- BC (OR=1.08, 95% CI=0.97-1.19, p=0.155), without heterogeneity. The detailed data was contained in **Table 2**.

### BC subtypes and smoking duration

This study grouped data by 10-year active smoking aimed to investigate the correlation between different smoking duration and BC subtype. The analysis showed that active smoking for less than 10 years did not increase the incidence of BC, regardless of whether it was ER+ BC (OR=0.99, p=0.870) or ER- BC (OR=1.02, p=0.699). Active smoking for more than 10 years had no effect on ER- BC (OR=1.08, p=0.105), but could increase the incidence of ER+ BC (OR=1.14, p=0.007). The detailed data was contained in **Table 2**.

### BC subtypes and smoking intensity

This study investigated the effect of smoking on BC subtypes at different smoking intensities by grouping data at 10 cigarettes per day boundaries. Subgroup analysis showed that smoking less than 10 cigarettes per day (OR=1.08, p=0.041) and more than 10 cigarettes per day (OR=1.18, p=0.002) could increase the risk of ER+ BC, and the risk was positively related to smoking intensity. For ER- BC, smoking less than 10 cigarettes per day had not been discovered as being effective (OR=0.97, p=0.603), However, smoking more than 10 cigarettes per day could increase the risk of suffering from ER- BC (OR=1.18, p=0.049). The results suggested that the occurrence of ER+ BC was more likely to be affected by active smoking than ER- BC. The detailed data was contained in **Table 2**.

### Overall effect of passive smoking

Thirty-nine studies documented BC risk data from passive smoking in women. The analysis showed that the risk of BC was significantly higher among women who passively smoked than those without passive smoking episode (OR=1.17, 95% CI=1.09-1.24, p<0.001, I^2 =^ 59.2%). Among them, current passive smoking had a significant effect on BC (OR=1.31, 95% CI=1.08-1.60, p=0.007, I^2 =^ 27.6%), but such history had no effect on BC (OR=1.18, 95% CI=0.97-1.43, p=0.107, I^2 =^ 42.5%). This suggests that passive smoking, especially current passive smoking would increase the risk of BC. Furthermore, cohort studies (OR=1.08, 95% CI=1.03-1.13) and case-control studies (OR=1.15, 95% CI=1.14-1.39) had consistently concluded that passive smoking increases the risk of BC in women. The detailed data was shown in **Table 3**.

**Table 3:** Effects of passive smoking on breast cancer incidence.

Subgroup analysis	No. ofstudies	OR	95%CI	*p*	Heterogeneity (I^2^) (%)
Ever passive smoking	39	1.17	1.09-1.24	*<0.001*	59.2
Current	4	1.31	1.08-1.60	*0.007*	27.6
Former	4	1.18	0.97-1.43	0.107	42.5
Cohort study	11	1.08	1.03-1.13	*0.002*	0
Case-control study	28	1.26	1.14-1.39	*<0.001*	66.5
Premenopausal BC	11	1.29	1.13-1.49	*<0.001*	37.3
Postmenopausal BC	11	1.13	0.93-1.36	0.218	73.5
Places exposed to passive smoking					
Home	11	1.07	0.95-1.21	0.269	63.2
Work	11	1.09	1.00-1.20	0.051	46.7
Home and work	5	1.40	1.00-1.97	0.051	88.3
Age stage exposure to passive smoking					
Childhood	16	1.15	1.05-1.25	*0.002*	63.7
Adult	15	1.21	1.04-1.40	*0.014*	79.1
Childhood and adult	8	1.49	1.15-1.93	*0.003*	72.2
Years passive smoked					
<10 years	15	0.99	0.89-1.10	0.876	8.4
10-20 years	19	1.13	1.03-1.25	*0.011*	41.2
20-30 years	17	1.38	1.18-1.61	*<0.001*	76.2
>30 years	9	1.35	1.10-1.65	*0.004*	74.4

OR, odd ratio; CI, confidence interval; BC, breast cancer.

### Menopausal status

Eleven studies included data on the relationship between passive smoking and BC in different menopausal states. The analysis showed that passive smoking increased the risk of premenopausal BC (OR=1.29, 95% CI=1.13-1.49, p<0.001, I^2 =^ 37.3%), but had no effect on the incidence of postmenopausal BC (OR=1.13, 95% CI=0.93-1.36, p=0.218, I^2 =^ 73.5%). The detailed data was contained in **Table 3**.

### Places exposed to passive smoking

Regarding the relationship of passive smoking and BC in different exposure places, 11 studies had data on home exposure, 11 studies had data on work exposure, and 5 studies had data on both home and work exposure. Subgroup analysis showed no relationship between passive smoking and BC incidence in different passive smoking exposure settings. However, passive smoking exposure at work (OR=1.09, p=0.051) and exposure at both home and work (OR=1.40, p=0.051) had a trend of harm to female population. The detailed data was contained in **Table 3**.

### Age stage exposure to passive smoking

In terms of the association between passive smoking and BC at different exposure ages, 16 studies had data on exposure in childhood, 15 studies had data on exposure in adult, and 8 studies had data on exposure in children and adult. Subgroup analyses showed that passive smoking increased BC risk regardless of exposure to childhood (OR=1.15, p=0.002), adult (OR=1.21, p=0.014), or both childhood and adult (OR=1.49, p=0.003). Among them, the increased risk of BC in those with simultaneous exposure in childhood and adult was significantly greater than that in those only with a single age group. The detailed data was contained in **Table 3**.

### Years passive smoked

Years were used to measure the duration of passive smoking exposure in this study. The relevant data were divided into ‘less than 10 years group’, ‘10-20 years group’, ‘20-30 years group’, and ‘more than 30 years group’, in the way most studies were segmented. This study showed that passive smoking which duration was less than 10 years in female population had no effect on BC (OR=0.99, p=0.876), while passive smoking exposure for 10-20 years (OR=1.13, p=0.011), 20-30 years (OR=1.38, p<0.001) and more than 30 years (OR=1.35, p=0.004) had a significant impact on the incidence of BC, compared to women who had never smoked. In all, increased incidence was positively correlated with longer duration of passive smoking exposure. The detailed data was contained in **Table 3**.

### Study quality

The NOS checklist was adopted to objectively evaluate the quality of included observational studies in this meta-study. 95.83% of the cohort studies were of high quality (NOS score >7), while 94.33% case-control studies were of high quality (NOS score >7). The quality ratings of cohort and case-control studies were listed in **Supplementary Tables 2** and **3**.

### Publication bias and sensitivity analysis

Publication bias was evaluated by the Begg’s test. The results of Begg’s test indicated the absence of publication bias among included articles (p>0.05). Sensitivity analysis was used to assess whether the individual studies affected the overall results or not. The results indicated that the analysis was relatively stable.

## Discussion

Through data analysis, this study found that smoking (active and passive) increases the risk of BC in women, with cohort and case-control studies showing consistent conclusions. Subgroup analysis of smoking-related factors showed that the effect of smoking on BC was positively correlated with smoking intensity and smoking duration. Among active smokers, current active smoking is more harmful to women than previous active smoking. With the increase of smoking cessation time, the harm of previous smoking history to the female population decreased. No differences were observed in the effect of smoking on BC at different starting ages. Among passive smokers, current passive smoking increases the incidence of BC, but past passive smoking does not. No differences in the effects of smoking on BC were observed between different passive smoking exposure sites and exposure age groups.

Subgroup analyses of population-related factors showed that smoking significantly increased the risk of BC in the multiparous population, but not in the nulliparous population. Smoking before the first birth has a greater effect on BC risk than smoking after the first birth. The risk of BC increases in women of different reproductive statuses with increasing duration of smoking.

Subgroup analysis of BC-related factors showed that smoking increases the risk of premenopausal BC, but has no effect on postmenopausal BC. At the same time, it can be clearly observed that smoking increases the risk of ER+ BC, and it is positively correlated with smoking duration and smoking intensity. For ER-BC, there was a trend of harm to women from smoking with increasing duration and intensity of smoking, but the difference did not reach statistical significance.

There is no consensus on the mechanism by which smoking increasing the risk of BC in women. The mainstream view is that smoking-specific DNA adducts (116, 117) (chemical carcinogens are activated by enzymes into electrophile and covalent combined with DNA, which are used to show DNA damage of specific carcinogens in human tissues (118)), mutations, and mal-regulated signaling pathways (119) represented by p53 [genes that inhibit cells from turning into cancer cells (75)] are the most important factors in BC (120). Animal and *in vitro* studies have shown that fat-soluble mutagenic compounds (121) in tobacco smoke, such as polycyclic hydrocarbons (122), aromatic amines (20) and N-nitrosamines (123), are major components of DNA adducts that can induce breast tumors (117) and have been detected in human milk (116). Compared with nonsmokers, detectable increases in cancer-causing DNA adducts were found in BC tissues and normal tissue adjacent to tumors in smokers (34, 124). In addition, studies have found that tobacco alters the incidence and spectrum of p53 mutations in breast cells, making smokers significantly more likely to carry p53 mutations (125). The potentially increased mutations affect related signaling pathways in smokers’ breast cells, hinder damage DNA repair and apoptosis, cause the body to be unable to respond to oncogenic signals, and ultimately induce tumors (126). The longer the exposure and the greater the intensity, the greater the effect (127). The starting point for these mechanisms is the compounds in tobacco smoke, which are present both in the smoke inhaled by smokers (mainly active smokers) and in the smoke exhaled by smokers and the end of lit cigarettes (mainly passive smokers) (128). This supports the conclusion in this study that both active smoking and passive smoking can induce BC in women, and confirms the biological plausibility of the positive correlation between BC risk and smoking intensity and duration. In addition, smoking status was correlated with the levels of carcinogenic DNA adducts in normal tissues adjacent to tumors, with a significant linear trend in the levels of carcinogenic DNA adducts in never-smokers, former smokers, and current smokers (19). When tobacco exposure was stopped, cancer cells became less active and the mutant gene was partially restored (34, 129). This supports our findings that the risk of current smoking is greater for women than previous smoking, and that the risk of BC from previous smoking decreases as the duration of cessation increases.

A relatively new view is that the harmful effects of smoking on BC depend on the antagonism of the estrogen-like and anti-estrogen-like effects of tobacco. According to previous studies, the health of the female breast is affected by the level and proportion of estrogen and progesterone (130, 131). Long-term exposure to estrogen or increased cell response to estrogen is an important risk factor for BC development (132, 133). On the one hand, carcinogenic metal-like metals in tobacco (106, 134), such as cadmium, chromium and arsenic, can induce estrogen receptor activation through hormone-binding domains and play estrogen-like roles in cell culture and animals (134). On the other hand, polycyclic aromatic hydrocarbons substances in tobacco play an anti-estrogen-like effect by competing with estrogen receptors or inducing hormone metabolism to reduce the level of active estrogen in the body (135, 136). At present, researchers tend to believe that the estrogen-like effect of tobacco and its carcinogenic effect are far superior to the breast protective effect brought by the anti-estrogen effect (114, 137). The anti-estrogen effect may cause breast cells to increase the number of estrogen receptors and enhance the sensitivity to estrogen, thus leading to the occurrence of hormone-sensitive tumors (138). There is accumulating evidence that ER+ and lobular BCs are more sensitive to ovarian hormones than are ER- and ductal cancers (139, 140). This may explain why smoking increases the incidence of ER+ BC, and the risk is positively correlated with the duration and intensity of smoking, but had no effect on ER- BC. In addition, premenopausal women have active gonadal function and secrete more estrogen (12, 129), which further aggravates the imbalance between estrogen and anti-estrogen effect on the basis of estrogen-like effect caused by tobacco, thus more likely to lead to the higher occurrence of BC (141). This supports the conclusion in this study that smoking increases the risk in premenopausal BC development, but not in postmenopausal BC development.

Based on the above two theories, tobacco exposure during the critical period is also considered to be an important factor affecting the occurrence of BC (100, 142). Animal models show that breast tissue is highly differentiated from puberty to the first full-term pregnancy, during which time the rapidly dividing cells are susceptible to malignant transformation due to carcinogens (143, 144). This period is therefore considered to be the period when tobacco smoke causes the greatest carcinogenic damage to breast tissue (145). During or after pregnancy, the second stage of BC carcinogenic damage is considered to be due to the onset of lactation, when breast cells are again active proliferation and vulnerable to tobacco smoke (146, 147). This may explain why smoking before the first birth had a greater impact on BC risk than smoking after the first birth. Unfortunately, no significant difference was observed in the subgroup analysis of the effect of smoking initiation on BC at different age in this study. In addition, increased exposure to estrogen (148), progesterone (149), and insulin-like growth factor (increased by growth hormone) (150) during pregnancy has been associated with promoting BC cell proliferation, which can trigger and/or promote tumors during continued tobacco exposure, known as “pregnancy-associated BC” (151–153). Epidemiological studies have found a higher incidence of BC in all multiparous women with, compared to all multiparous women regardless of their age (154–157). The higher incidence rate of BC in the multiparous population and the impact of tobacco exposure on estrogen levels in pregnant people may explain why smoking significantly increases the risk of BC in the multiparous population, but had no impact in the nulliparous population.

According to the above mechanisms and the characteristics of different included studies, we believe that the reasons for the differences between different studies may be as follows: First, each study has different assessment methods for exposure factors. Questionnaires and interviews both produce recall bias. The rigor of questionnaire design and the professionalism of interviewers will affect the validity of data collection, which makes researchers inevitably biased when exploring the relationship between smoking and BC; second, The duration of follow-up in the included studies varied considerably. The occurrence of BC often takes years to decades, and there is no exact number of years, but a longer follow-up period can often find more cases of BC, which can provide more abundant research data, conversely, a shorter follow-up period Time, not only limited the researchers’ discovery of the association between smoking and BC, but also prevented subgroup analyses; third, different studies defined smoking differently. According to World Health Organization (WHO) regulations, people who smoke continuously or cumulatively for 6 months or more are smokers in some studies, some studies extend the duration to 1 year, and some studies define smokers as long as they smoke. Different criteria make the baseline status of the control population different, and although the concentration of carcinogens in tobacco is not high, it may still have an impact on the final results with long-term follow-up. Therefore, we believe that the results of the study can be improved by shortening the time between two follow-up visits, increasing the number of follow-up visits, and updating them in a timely manner. In addition, large-scale cohort studies are still a feasible way to verify the conclusions of this study and narrow the differences between different studies.

Reviewing the same type of studies, A-sol Kim et al.’s study (158) reached a similar conclusion to the present study that passive smoking increases the risk of BC in women (OR=1.23, 95%CI=1.10-1.38). However, they did not perform subgroup analysis on population and smoking factors, thus could not provide reference to the female population from multiple aspects. Moreover, they only included those who had never smoked, did not consider those who had previously smoked and had successfully gone through smoking cessation. These may have led to their findings being overestimated and lacking reliability. The study by Lisa A DeRoo et al (159) did not find any association between smoking and BC. This may be due to the limited number of studies they included, or it may be that the low concentration of carcinogens in tobacco with a long latency to harm the breast make the relationship between smoking and BC not easily observed.

While this meta-analysis yielded comprehensive and objective conclusions, there were still some potential limitations to consider. Firstly, the design, study population, sample size, risk assessment, and adjustment for related confounding factors varied among the included studies, which may bias the results and reduce the confidence of the conclusions. Therefore, this study used a random-effects model to evaluate the effect of smoking on BC. Secondly, most studies used questionnaires to assess smoking exposure, and a few used the form of interviews or a combination of interviews and questionnaires, therefore inevitably led to evaluation bias or recall bias during the evaluation, especially the case-control studies nested in the cohort, which may bias the findings. Therefore, this study selected relevant data adjusted for the largest number of potential confounders for statistical analysis to improve the accuracy of the conclusions. Thirdly, some trials did not report more adequate subgroup data, such as BC type subgroup data, fertility status subgroup data, etc., which made it very difficult to conduct some subgroup analyses in this study.

Apart from its limitations, this meta-analysis had its own strengths. Firstly, this study included a large number of observational studies including more than 2.3 million participants in Asia, Europe, America, and Oceania. The larger observational population increases the reliability and authenticity of the conclusions of this study. Additionally, this study grouped the extracted data (by smoking related factors, population related factors, BC-related factors) and performed subgroup analysis to comprehensively explore the possibility of the effect of different kinds of smoking on different populations, different BC types from different aspects. Overall, this meta-analysis led to some meaningful conclusions that may provide a new reference for BC prevention in the female population.

## Conclusion

This meta-analysis found that smoking (active and passive smoking) increases the risk of BC in the female population, especially premenopausal BC and ER+ BC, but had no effect on postmenopausal BC and ER- BC. The risk of BC was positively associated with the longer duration and stronger intensity of smoking, negatively associated with the duration of smoking cessation. Smoking increases BC risk in the multiparous population, but had no effect in the nulliparous population, where smoking before the first birth had a larger effect on BC risk than smoking after the first birth.

## Data availability statement

The original contributions presented in the study are included in the article/**Supplementary Material**. Further inquiries can be directed to the corresponding author.

## Author contributions

All authors helped to perform the research. YH and XL writing manuscript; YH and YS performing procedures and data analysis; JH and CY contribution to writing the manuscript; NH contribution to drafting conception and design. All authors contributed to the article and approved the submitted version.

## Conflict of interest

The authors declare that the research was conducted in the absence of any commercial or financial relationships that could be construed as a potential conflict of interest.

## Publisher’s note

All claims expressed in this article are solely those of the authors and do not necessarily represent those of their affiliated organizations, or those of the publisher, the editors and the reviewers. Any product that may be evaluated in this article, or claim that may be made by its manufacturer, is not guaranteed or endorsed by the publisher.

## Abbreviations

BC, breast cancer; MOOSE, the meta-analysis of observational studies in epidemiology; PICOS, the population, intervention, comparison, outcome and setting criteria; NOS, the newcastle-ottawa quality assessment scale checklist; ER+, estrogen receptor-positive; ER-, estrogen receptor-negative; CI, confidence interval; OR, odd ratio; BMI, body mass index; WHO, world health organization.
