# Supplementary material for: The relationship between tobacco and breast cancer incidence: A systematic review and meta-analysis of observational studies
Source: Front Oncol. 2022 Sep 15;12:961970. doi: 10.3389/fonc.2022.961970 (PMC9520920; doi:10.3389/fonc.2022.961970)
Supplement: Supplementary file 1 [file Table_1.docx]

**Supplementary Table 1**. Characteristics of included observational studies in the meta-analysis

| Author, year | Number of smokers (include active and passive) | Number of non-smokers | Time of experiment (year) | Study | Population | Exposure assessment | Adjusting parameters |
| --- | --- | --- | --- | --- | --- | --- | --- |
| Vatten LJ, 1990 | 24,466 | 151 | 1974-1978 | NA | men and women | questionnaire | age at entry, age at diagnosis, occupational status and BMI |
| Bennicke K, 1995 | 1,828 | 1,412 | 1989-1991 | NA | women | interview | age |
| Calle EE, 1994 | 319,446 | 254,122 | 1982-1988 | The Cancer Prevention Study II | women | questionnaire, interview | family history of BC in mother or sisters, a personal history of breast cysts, BMI, years of education, alcohol use, age at first birth, age at menarche, age at menopause, and consumption of vegetables and high-fiber grains and total dietary fat |
| Goodman MT, 1997 | 12,210 | 9,990 | 1979-1981 | The Life Span Study | women | questionnaire | city, attained age, age at the time of the bombings, and radiation dose to the breast |
| Nishino Y, 2001 | 3,917 | 5,758 | 1984-1992 | NA | men and women | questionnaire | age, study area, alcohol drinking, green and yellow vegetable intake, miso-soup intake, pickled vegetable intake, meat intake for colon cancer and rectal cancer, meat intake and past history of lung disease for lung cancer, age at first birth, number od live births, age at menarche, and BMI for BC |
| Hanaoka T, 2005 | 15,630 | 6,175 | 1990-1999 | The Japan Public Health Center Study | women | questionnaire | public health center, age, employment status, education level, BMI, family history of BC in mother or sisters, history of past benign breast disease, age at menarche, number of births, menopausal status, hormone use and alcohol consumption per week |
| Olson JE, 2005 | 35,789 | 1,316 | 1986-1999 | The Iowa Women’s Health Study | women | questionnaire | age, education, family history of BC, age at menarche, age at menopause, oral contraceptive use, hormone replacement therapy, BMI, waist-to-hip ratio, height, BMI at the age of 18 years, physical activity, and alcohol consumption |
| Lin Y, 2005 | 2,387 | 32,023 | 1988-2001 | The Japan Collaborative Cohort Study | women | questionnaire, interview | age, area, BMI, family history of BC, alcohol drinking, daily walking, age at menarche, age at the birth of her first child, menopause status at baseline, number of births, and use of sex hormons |
| Pirie K, 2008 | 174,819 | 35,828 | 1996-2001 | The Million Women Study | women | questionnaire | age,region of residence, socio-economic group, age at menarche, parity,age at first birth, menopausal status, BMI, physical activity, alcohol consumption and use of hormonal therapy for the menopause |
| Reynolds P, 2009 | 50,453 | 7,070 | 1995-2007 | The California Teachers Study | women | questionnaire, interview | family history of BC, age at menarche, pregnancy history, lifetime duration of breast-feeding, physical activity, alcohol consumption, BMI, and categories of menopausal status with use of hormone therapy |
| Xue F, 2010 | 85,683 | 36,017 | 1976-2006 | The Nurses' Health Study | women | questionnaire | age, family history of BC, age at menarche, height, BMI at age 18 years, oral contraceptive use, history of benign breast disease, leisure-time physical activity, alcohol consumption, passive smoking at home and at work, age at first birth, parity, current BMI, age at menopause, menopausal status, and postmenopausal hormone use |
| Luo J, 2011 | 40,600 | 39,300 | 1998-2009 | The Women's Health Initiative Observational Study | women | questionnaire | age at enrolment, ethnicity, education, BMI, physical activity, alcohol intake, parity, family history of BC, hormone therapy use, age at menarche, and age of first live birth |
| Rosenberg L, 2013 | 50,975 | 8,025 | 1995-2011 | The Black Women's Health Study | women | questionnaire | age, questionnaire cycle, BMI, years of education, family history of BC, age at menarche, parity, age at first birth, oral contraceptive use, vigorous exercise, alcoholic beverage consumption, menopausal status, age at menopause, and menopausal female hormone use |
| Dossus L, 2014 | 183,608 | 139,380 | 2000-2012 | The European Prospective Investigation into Cancer and Nutrition Study | women | interview | BMI at recruitment, educational level, ever use of oral contraceptives, ever use of menopausal hormone therapy, menopausal status at baseline, parity and age at first full-term pregnancy, age at menarche, alcohol consumption, physical activity |
| Catsburg C, 2015 | 44,020 | 45,815 | 1980-2013 | The Canadian National Breast Screening Study | women | questionnaire | age at enrollment, randomization group, study center, BMI, education level, vigorous physical activity, use of oral contraceptives, use of hormone therapy, number of live births, age at menarche, family history of BC in first-degree relatives, history of benign breast disease, menopausal status, practice of breast self-examination and alcohol consumption |
| Wada K, 2015 | 14,214 | 1,505 | 1992-2008 | The Takayama study | women | questionnaire | age, BMI, physical activity score, alcohol consumption, education years, age at menarche, age at first delivery, menopausal status, parity number and history of hormone replacement therapy |
| White AJ, 2017 | 22,401 | 28,483 | 2003-2014 | The National Institute of Environmental Health Sciences Sister Study | women | questionnaire, interview | age, race, education, age at menarche, age at first birth, parity, use of hormonal birth control, use of postmenopausal hormones, age at menopause and menopausal status and BMI |
| van den Brandt PA, 2017 | 24,592 | 37,981 | 1986-2016 | The Netherlands Cohort Study | women | questionnaire | age at baseline, current smoking status at baseline, body height, BMI, non-occupational physical activity, highest level of education, family history of BC in mother or sisters, history of benign breast disease, age at menarche, parity), age at first birth, oral contraceptive use, post_x005f menopausal HRT, passive smoking status at work, at home and parental, nutritional supplement use, and alcohol intake |
| Jones ME, 2017 | 90,062 | 12,865 | 2003-2017 | The Generations Study cohort | women | questionnaire | attained age, time since recruitment to cohort, birth cohort, benign breast disease, family history of BC in first-degree relatives, socio-economic score, age at menarche, age at first pregnancy, parity, duration of breastfeeding, current oral contraceptive use before menopause, alcohol consumption, physical activity, pre-menopausal BMI at age 20 years, post-menopausal BMI, menopausal hormone therapy use, menopausal status and age at menopause |
| Gram IT, 2019 | 65,010 | 2,303 | 1993-2013 | The Multiethnic Cohort study | women | questionnaire | age at cohort entry, race/ethnicity where applicable, BMI, family history of BC, age at first birth, number of children, age at menarche, age at and type of menopause, hormone replacement therapy, alcohol intake and education |
| Heberg J, 2019 | 10,835 | 5,271 | 1993-2016 | The Danish Nurse Cohort | women | questionnaire | alcohol intake, BMI, physical activity, self-rated health, cohabitation, parity, hormone replacement therapy, oral contraceptive use, number of births, age at menarche |
| Zeinomar N, 2019 | 7,277 | 10,157 | 1994-2018 | The Prospective Family Study Cohort | women | questionnaire | study site, race/ethnicity, BMI, education, and hormonal birth control use |
| Botteri E, 2021 | 15,910 | 14,020 | 1991-2012 | The Swedish Women's Lifestyle and Health Cohort Study | women | questionnaire | age, menopausal status, education |
| Gram IT, 2022 | 30,471 | 45,923 | 1991-2018 | The Norwegian Women and Cancer Study | women | questionnaire | age, duration of education, hormone therapy, age at menarche, family history of BC, age at first birth, number of children, hormonal contraceptive use, menopausal status, alcohol consumption and BMI |
| Kato I, 1992 | 1,058 | 758 | 1990-1991 | NA | women | questionnaire | the number of female family members and their ages |
| Field NA, 1992 | 1,864 | 1,376 | 1982-1984 | NA | women | questionnaire | birth year and county of residence based on logistic regression estimates |
| Pawlega J, 1992 | 116 | 261 | 1987-1990 | NA | women | questionnaire | age, education, social class, marital status, number of persons in household, BMI and drinking of vodka 20 years earlier |
| Chu SY, 1990 | 4,602 | 3,749 | 1981-1982 | The Centers for Disease Control Cancer and Steroid Hormone Study | women | questionnaire | age, parity, menopausal status, age at first birth, age at menarche, family history of BC, history of benign breast disease, and estrogen replacement therapy |
| Schechter MT, 1989 | 548 | 513 | 1982-1985 | NA | women | questionnaire | dietary fat consumption and alcohol intake |
| Adami HO, 1988 | 604 | 345 | 1984-1985 | NA | women | interview | education, age at menarche, age at first full-term pregnancy, parity, menopause, history of operation for benign breast disease, family history of BC, total duration of oral contraceptive use and alcohol consumption |
| Hirose K, 1995 | 11,315 | 13,034 | 1988-1992 | NA | women | questionnaire | age at menarche, age at first full-term pregnancy, parity, menopause |
| Smith SJ, 1994 | 799 | 703 | 1982-1985 | NA | women | questionnaire | age at menarche, nulliparity, age at first full-term pregnancy, breastfeeding, family history of BC, total oral contraceptive use, biopsy for benign breast disease, total alcohol consumption at age 18 |
| Braga C, 1996 | 1,678 | 3,479 | 1991-1994 | NA | women | questionnaire | age, centre, education, parity menopausal status/age at menopause, age at menarche, history of benign breast disease, family history of BC,use of oral contraceptives and BMI |
| Ranstam J, 1955 | NA | NA | 1980-1984 | NA | women | questionnaire | menopausal status, age at first term pregnancy, age at menarche, family history of BC in first degree relatives, duration of oral contraceptive use, BMI, alcohol intake, socioeconomic status |
| Morabia A, 1998 | 1,050 | 251 | 1992-1993 | NA | women | questionnaire | age |
| Tung HT, 1999 | 215 | 591 | 1990-1995 | NA | women | questionnaire | age at diagnosis |
| Johnson KC, 2000 | 2,463 | 2,292 | 1994-1997 | NA | women | questionnaire | 10-year age groups, province, education, BMI, alcohol use, age at menarche, age at first pregnancy 5 months or longer, number of live births, months of breastfeeding, physical activity and height |
| Marcus PM, 2000 | 786 | 868 | 1993-1996 | The Carolina BC Study | women | questionnaire | race and age at diagnosis/selection |
| Ueji M, 1998 | 43 | 342 | 1990-1997 | NA | women | questionnaire | family history of BC, education, age at menarche, age at primiparity, parity |
| Lash TL, 2002 | 1,102 | 179 | 1987-1993 | NA | women | questionnaire, interview | history of medical radiation therapy, BMI, family history of BC, history of BC, history of benign breast disease, frequency of alcoholic drinks, age at first birth, and parity |
| Kropp S, 2002 | 1,373 | 188 | 1999-2000 | NA | women | questionnaire | average daily alcohol intake, total number of months of breastfeeding, education, first degree family history of BC, menopausal status, and BMI |
| Liu L, 2000 | 174 | 198 | 1994-1996 | NA | women | questionnaire | BMI,history of BC, history of benign breast disease, frequency of alcoholic drinks |
| Shrubsole MJ, 2004 | 1,770 | 360 | 1996-1998 | The Shanghai BC Study | women | interview | age, education, family history of BC, personal history of fibroadenoma, age at menarche, parity, age at first live birth, menopausal status, age at menopause, physical activity and waist-to-hip ratio |
| Alberg AJ, 2004 | 157 | 66 | 1989-1995 | NA | women | interview | age and menopausal status |
| Gammon MD, 2004 | 2,414 | 325 | 1996-1998 | The Long Island BC Study Project | women | questionnaire | age, history of benign breast disease, BMI at age 20, family history of BC, history of fertility problems, number of pregnancies, menopausal status, and weight in year prior to reference date |
| Manjer J, 2004 | 414 | 387 | 1994-1999 | The Malmo Diet and Cancer Study | women | questionnaire | age at baseline, storage time and sub-cohort |
| Bonner MR, 2005 | NA | NA | 1998-2003 | The Western New York Exposures and BC Study | women | questionnaire | age, education, race, previous benign breast disease, parity, age at menarche, BMI, age at first birth, relative with BC, total alcohol consumption and age at menopause for post-menopausal women |
| Metsola K, 2005 | 573 | 392 | 1990-1995 | NA | women | questionnaire | age, age at menarche, age at first full term pregnancy, number of pregnancies, history of benign breast disease, first degree family history of BC, weist-to-hip ratio, smoking and use of alcohol |
| Mechanic LE, 2006 | 3,478 | 855 | 1999-2005 | NA | women | questionnaire | offsets, age, age at menarche, age at first full-term pregnancy/parity composite, family history and alcohol |
| Ha M,2007 | 12,078 | 294 | 1989-2005 | The US Radiologic Technologists study | women | questionnaire | alcohol intake, age at menarche, age at first childbirth, parity, family history of BC, hormone replacement therapy, year that a woman first worked as a radiologic technologist, BMI, and menopausal status |
| Roddam AW, 2007 | 672 | 607 | 1998-2006 | NA | women | questionnaire | socioeconomic status, alcohol consumption, BMI, parity and age at first giving birth, use of oral contraceptives, family history of BC, age at menarche, menopausal status |
| Slattery ML,2008 | 1,126 | 1,140 | 2005-2007 | NA | women | questionnaire | age, center, BMI, aspirin/NSAIDs, parity, long-term alcohol use, long-term physical activity, and recent estrogen in menopausal women |
| Rollison DE, 2008 | 352 | 246 | 2000-2002 | NA | women | questionnaire | age, education, and menopausal status |
| Young E, 2009 | 10,100 | 2,668 | 2002-2007 | The Ontario Women's Health Study | women | questionnaire | age |
| Ahern TP, 2009 | 847 | 142 | 2005-2008 | NA | women | interview | reference age, menopausal status, pack-years of active cigarette smoking, BMI at interview, parity, average grams of alcohol consumed per day from age 30 to 39, and family history of BC |
| Conlon MS, 2010 | 1,068 | 54 | 2002-2004 | NA | women | questionnaire | age |
| De Silva M,2010 | NA | NA | 2007-2009 | NA | women | questionnaire | BMI, use of oral contraceptives, use of hormone therapy, number of live births, age at menarche, family history of BC in first-degree relatives, history of benign breast disease, menopausal status, alcohol consumption |
| Sezer H, 2011 | 125 | 430 | 2010-2011 | NA | women | interview | age |
| Hu M, 2013 | 19 | 388 | 2009-2011 | NA | women | questionnaire, interview | age, physical activity, menopausal status, passive smoking, alcohol, BMI, family history of female cancer |
| Gao CM, 2013 | 21 | 1,330 | 2004-2007 | NA | women | questionnaire, interview | age, menopausal status, educational status, occupation, BMI and income/month |
| McKenzie F, 2013 | 2,302 | 2,037 | 2005-2007 | The New Zealand BC Study | women | questionnaire | age, menopause status, age at menarche, BMI, exercise, hormone replacement therapy, oral contraceptive, maternal BC, parity, alcohol, and socioeconomic position |
| Ilic M, 2013 | 128 | 254 | 2004-2005 | NA | women | questionnaire | educational level, marital status, age at menarche, menopausal status, breastfeeding history, family history of BC, BMI, alcohol use, and cardiovascular disease in personal medical history |
| Kawai M, 2014 | 1,057 | 1,801 | 2004-2010 | NA | women | questionnaire | age, reference year, and age at first live birth |
| Tong JH, 2014 | 335 | 288 | 2009-2010 | NA | women | interview | age at interview, age at menarche, menopausal status, oral contraceptive use, family history of cancer, alcohol consumption and BMI |
| Pimhanam C, 2014 | NA | NA | 2007-2011 | NA | women | questionnaire | BMI, history of biopsy, number of breast biopsy, history of breastfeeding, history of oral contraceptive usage, family history of BC, regularly exercise, and regularly eat grilled food |
| Li B, 2015 | 937 | 830 | 2007-2013 | NA | women | interview | age, residence, study stage, BMI, physical activity, age at menarche, age at first live birth, age at menopause, mother/sister/daughter with BC and history of benign breast disease |
| Connor AE, 2015 | 2,732 | 5,185 | 2004-2013 | The BC Health Disparities Study | women | questionnaire | age, study, history of alcohol consumption, menopausal status, parity, education, family history of BC,and BMI at referent year |
| Hara A, 2017 | 213 | 825 | 2010-2011 | NA | women | questionnaire | age, BMI, number of children, menopausal status, family history of BC, and habitual drinking |
| Butler EN, 2016 | 1,590 | 1,782 | 2001-2015 | The Carolina BC Study | women | questionnaire,interview | age, race, family history, alcohol use, menopausal status, oral contraceptive use, parity, age at first birth, age at first breastfeeding, age at menarche, BMI, and offsets |
| Park SY, 2016 | 5,791 | 17,376 | 1993-2013 | The African American BC Epidemiology and Risk Consortium | women | questionnaire | education, age at menarche, age at first birth, parity, status of and age at menopause, oral contraceptive use, estrogen only use, estrogen and progesterone use, BMI, family history of BC and alcohol consumption |
| Strumylaite L, 2017 | 585 | 794 | 2007-2011 | NA | women | questionnaire | age, number of births, estrogen-active period, hormone therapy during menopause, family history of BC, alcohol use, BMI, education, marital status, diabetes mellitus, and thyroid diseases |
| Dianatinasab M, 2017 | NA | NA | 2014-2016 | NA | women | questionnaire | education, occupation, family history of BC, oral contraceptive usage, chest X-ray, hair coloring, physical exercise, BMI, birth weight, marital age, age at first delivery, parity, birth interval, breastfeeding, menarche age, menopause status, stressful life, sleep quality and regular bedtime |
| Ellingjord-Dale M, 2017 | 26,916 | 1,784 | 2006-2014 | The Norwegian BC Screening Program | women | questionnaire | physical activity and alcohol |
| Regev-Avraham Z, 2018 | 297 | 114 | 2008-2013 | NA | women | questionnaire, interview | age at interview and BMI |
| Godinho-Mota JCM, 2019 | 179 | 363 | 2008-2014 | NA | women | questionnaire | age |
| Alsolami FJ, 2019 | 41 | 391 | 2014-2016 | NA | women | questionnaire | family history of BC, oral contraceptive usage, physical exercise, BMI, birth weight, marital age, age at first delivery, parity, birth interval, breastfeeding, menarche age, menopause status |
| Baset Z, 2021 | 68 | 334 | 2018-2019 | NA | women | questionnaire | age and BMI |

NA: not available; BC: breast cancer; BMI: body mass index; HRT: hormone replacement therapy
