# Supplementary material for: The relationship between tobacco and breast cancer incidence: A systematic review and meta-analysis of observational studies
Source: Front Oncol. 2022 Sep 15;12:961970. doi: 10.3389/fonc.2022.961970 (PMC9520920; doi:10.3389/fonc.2022.961970)
Supplement: Supplementary file 2 [file Table_2.docx]

**Supplementary Table 2.** Quality assessment of cohort studies included.

| Author, year,  Study (Observational) | **Selection (Out of 4)** | | | | **Comparability**  **(Out of 2)** | **Outcomes (Out of 3)** | | | **Total**  **(Out of 9)** |
| --- | --- | --- | --- | --- | --- | --- | --- | --- | --- |
|  | Representativeness of exposed cohort | Selection of non exposed cohort | Ascertainment  of exposure | Outcome not present at the start of the study |  | Assessment of outcomes | Length of follow-up | Adequacy of follow up of cohorts |  |
| Vatten LJ, 1990 | 1 | 0 | 1 | 1 | 2 | 1 | 1 | 1 | 8 |
| Bennicke K, 1995 | 1 | 1 | 1 | 1 | 1 | 1 | 1 | 1 | 8 |
| Calle EE, 1994 | 1 | 1 | 0 | 1 | 1 | 1 | 1 | 1 | 7 |
| Goodman MT, 1997 | 1 | 1 | 1 | 1 | 2 | 1 | 1 | 1 | 9 |
| Nishino Y, 2001 | 1 | 1 | 1 | 0 | 2 | 1 | 1 | 1 | 8 |
| Hanaoka T, 2005 | 1 | 1 | 1 | 1 | 2 | 1 | 1 | 0 | 8 |
| Olson JE, 2005 | 0 | 1 | 1 | 1 | 2 | 0 | 1 | 1 | 7 |
| Lin Y, 2005 | 1 | 1 | 0 | 1 | 2 | 1 | 1 | 1 | 8 |
| Pirie K, 2008 | 1 | 1 | 1 | 1 | 2 | 1 | 1 | 1 | 9 |
| Reynolds P, 2009 | 1 | 0 | 1 | 1 | 2 | 1 | 1 | 1 | 8 |
| Xue F, 2010 | 1 | 1 | 1 | 1 | 2 | 1 | 1 | 1 | 9 |
| Luo J, 2011 | 1 | 0 | 1 | 1 | 1 | 1 | 0 | 1 | 6 |
| Rosenberg L, 2013 | 1 | 1 | 1 | 1 | 2 | 1 | 1 | 1 | 9 |
| Dossus L, 2014 | 1 | 1 | 0 | 1 | 2 | 1 | 1 | 1 | 8 |
| Catsburg C, 2015 | 0 | 1 | 1 | 1 | 2 | 1 | 1 | 0 | 7 |
| Wada K, 2015 | 1 | 1 | 1 | 1 | 1 | 1 | 1 | 1 | 8 |
| White AJ, 2017 | 1 | 1 | 1 | 1 | 2 | 1 | 1 | 1 | 9 |
| van den Brandt PA, 2017 | 1 | 1 | 1 | 0 | 2 | 1 | 1 | 1 | 8 |
| Jones ME, 2017 | 1 | 1 | 0 | 1 | 2 | 1 | 1 | 1 | 8 |
| Gram IT, 2019 | 1 | 1 | 1 | 1 | 2 | 1 | 1 | 1 | 9 |
| Heberg J, 2019 | 0 | 1 | 1 | 1 | 2 | 1 | 0 | 1 | 7 |
| Zeinomar N, 2019 | 1 | 1 | 1 | 0 | 2 | 1 | 1 | 1 | 8 |
| Botteri E, 2021 | 1 | 1 | 1 | 1 | 2 | 0 | 1 | 1 | 8 |
| Gram IT, 2022 | 1 | 1 | 1 | 1 | 2 | 1 | 1 | 1 | 9 |

The observational studies were assessed by the Newcastle-Ottawa Quality Assessment Scale (NOS) checklist of cohort studies.
