# Supplementary material for: The relationship between tobacco and breast cancer incidence: A systematic review and meta-analysis of observational studies
Source: Front Oncol. 2022 Sep 15;12:961970. doi: 10.3389/fonc.2022.961970 (PMC9520920; doi:10.3389/fonc.2022.961970)
Supplement: Supplementary file 3 [file Table_3.docx]

**Supplementary Table 3.** Quality assessment of case-control studies included.

| Author, year,  Study (Observational) | **Selection (Out of 4)** | | | | **Comparability**  **(Out of 2)** | **Outcomes (Out of 3)** | | | **Total**  **(Out of 9)** |
| --- | --- | --- | --- | --- | --- | --- | --- | --- | --- |
|  | Adequate case definition | Representativeness of the cases | Selection of controls | Definition of controls |  | Ascertainment of exposure | Same method of ascertainment for cases and controls | Non-response rate |  |
| Kato I, 1992 | 1 | 0 | 1 | 1 | 1 | 1 | 1 | 0 | 6 |
| Field NA, 1992 | 1 | 1 | 1 | 1 | 2 | 0 | 1 | 1 | 8 |
| Pawlega J, 1992 | 1 | 0 | 1 | 1 | 2 | 1 | 1 | 0 | 7 |
| Chu SY, 1990 | 1 | 0 | 1 | 1 | 2 | 1 | 1 | 0 | 7 |
| Schechter MT, 1989 | 1 | 1 | 0 | 1 | 2 | 1 | 1 | 1 | 8 |
| Adami HO, 1988 | 1 | 1 | 1 | 1 | 2 | 1 | 1 | 1 | 9 |
| Hirose K, 1995 | 1 | 0 | 1 | 0 | 2 | 1 | 1 | 1 | 7 |
| Smith SJ, 1994 | 1 | 1 | 1 | 1 | 2 | 1 | 1 | 1 | 9 |
| Braga C, 1996 | 0 | 1 | 1 | 1 | 2 | 0 | 1 | 0 | 6 |
| Ranstam J, 1955 | 1 | 0 | 1 | 1 | 2 | 1 | 1 | 1 | 8 |
| Morabia A, 1998 | 1 | 1 | 1 | 1 | 1 | 1 | 1 | 1 | 8 |
| Tung HT, 1999 | 1 | 1 | 1 | 1 | 2 | 1 | 1 | 1 | 9 |
| Johnson KC, 2000 | 1 | 1 | 1 | 1 | 2 | 1 | 1 | 1 | 9 |
| Marcus PM, 2000 | 1 | 0 | 1 | 1 | 2 | 1 | 1 | 1 | 8 |
| Ueji M, 1998 | 1 | 1 | 1 | 1 | 1 | 1 | 1 | 1 | 8 |
| Lash TL, 2002 | 1 | 1 | 1 | 1 | 2 | 1 | 1 | 1 | 9 |
| Kropp S, 2002 | 1 | 0 | 1 | 0 | 2 | 0 | 1 | 1 | 6 |
| Liu L, 2000 | 1 | 1 | 1 | 1 | 2 | 1 | 1 | 1 | 9 |
| Shrubsole MJ, 2004 | 0 | 1 | 1 | 1 | 1 | 1 | 1 | 1 | 7 |
| Alberg AJ, 2004 | 1 | 1 | 1 | 1 | 2 | 1 | 0 | 1 | 8 |
| Gammon MD, 2004 | 1 | 1 | 1 | 1 | 1 | 1 | 1 | 1 | 8 |
| Manjer J, 2004 | 1 | 1 | 1 | 1 | 1 | 1 | 1 | 1 | 8 |
| Bonner MR, 2005 | 1 | 1 | 1 | 1 | 2 | 1 | 1 | 0 | 8 |
| Metsola K, 2005 | 1 | 0 | 0 | 1 | 2 | 1 | 1 | 1 | 7 |
| Mechanic LE, 2006 | 1 | 1 | 1 | 1 | 1 | 1 | 1 | 1 | 8 |
| Ha M,2007 | 1 | 1 | 1 | 1 | 2 | 1 | 1 | 1 | 9 |
| Roddam AW, 2007 | 1 | 1 | 0 | 1 | 2 | 1 | 1 | 1 | 8 |
| Slattery ML,2008 | 1 | 1 | 1 | 1 | 2 | 1 | 1 | 1 | 9 |
| Rollison DE, 2008 | 1 | 1 | 1 | 1 | 1 | 0 | 1 | 1 | 7 |
| Young E, 2009 | 0 | 0 | 1 | 1 | 2 | 1 | 1 | 1 | 7 |
| Ahern TP, 2009 | 1 | 1 | 1 | 1 | 2 | 1 | 1 | 1 | 9 |
| Conlon MS, 2010 | 1 | 1 | 1 | 1 | 2 | 0 | 1 | 1 | 8 |
| De Silva M,2010 | 1 | 1 | 1 | 1 | 2 | 1 | 0 | 1 | 8 |
| Sezer H, 2011 | 1 | 1 | 1 | 0 | 2 | 1 | 1 | 1 | 8 |
| Hu M, 2013 | 1 | 1 | 1 | 1 | 2 | 1 | 0 | 1 | 8 |
| Gao CM, 2013 | 1 | 0 | 1 | 1 | 2 | 1 | 1 | 1 | 8 |
| McKenzie F, 2013 | 1 | 1 | 1 | 1 | 2 | 1 | 1 | 0 | 8 |
| Ilic M, 2013 | 1 | 1 | 1 | 1 | 2 | 0 | 1 | 1 | 8 |
| Kawai M, 2014 | 0 | 0 | 1 | 1 | 2 | 1 | 1 | 1 | 7 |
| Tong JH, 2014 | 1 | 1 | 1 | 1 | 1 | 1 | 1 | 1 | 8 |
| Pimhanam C, 2014 | 1 | 0 | 1 | 1 | 2 | 1 | 1 | 1 | 8 |
| Li B, 2015 | 1 | 1 | 1 | 1 | 2 | 1 | 1 | 1 | 9 |
| Connor AE, 2015 | 1 | 1 | 1 | 1 | 2 | 1 | 1 | 1 | 9 |
| Hara A, 2017 | 1 | 1 | 1 | 0 | 1 | 1 | 1 | 1 | 7 |
| Butler EN, 2016 | 1 | 0 | 1 | 1 | 2 | 1 | 1 | 1 | 8 |
| Park SY, 2016 | 1 | 1 | 1 | 1 | 2 | 1 | 1 | 1 | 9 |
| Strumylaite L, 2017 | 1 | 1 | 1 | 1 | 1 | 1 | 0 | 1 | 7 |
| Dianatinasab M, 2017 | 0 | 1 | 1 | 0 | 2 | 1 | 1 | 1 | 7 |
| Ellingjord-Dale M, 2017 | 1 | 1 | 1 | 1 | 2 | 1 | 1 | 0 | 8 |
| Regev-Avraham Z, 2018 | 1 | 1 | 0 | 1 | 2 | 1 | 1 | 1 | 8 |
| Godinho-Mota JCM, 2019 | 1 | 1 | 1 | 1 | 2 | 1 | 0 | 1 | 8 |
| Alsolami FJ, 2019 | 1 | 1 | 1 | 1 | 1 | 0 | 1 | 1 | 7 |
| Baset Z, 2021 | 1 | 1 | 1 | 1 | 2 | 1 | 1 | 1 | 9 |

The observational studies were assessed by the Newcastle-Ottawa Quality Assessment Scale (NOS) checklist of case-control studies.
